# Poly (Ether-Ether-Ketone) for Biomedical Applications: From Enhancing Bioactivity to Reinforced-Bioactive Composites—An Overview

**DOI:** 10.3390/polym15020373

**Published:** 2023-01-10

**Authors:** Mônica Rufino Senra, Maria de Fátima Vieira Marques, Sergio Neves Monteiro

**Affiliations:** 1 Instituto de Macromoleculas Professor Eloisa Mano, Universidade Federal do Rio de Janeiro, Horácio Macedo Av., 2.030, Bloco J, Cidade Universitária, Rio de Janeiro CEP 21941-598, RJ, Brazil; 2 Department of Materials Science, Military Institute of Engineering, IME, Praça General Tibúrcio, 80, Urca, Rio de Janeiro CEP 22290-270, RJ, Brazil

**Keywords:** PEEK, surface modification, bioactive composites, bone implants

## Abstract

The global orthopedic market is forecasted to reach US$79.5 billion by the end of this decade. Factors driving the increase in this market are population aging, sports injury, road traffic accidents, and overweight, which justify a growing demand for orthopedic implants. Therefore, it is of utmost importance to develop bone implants with superior mechanical and biological properties to face the demand and improve patients’ quality of life. Today, metallic implants still hold a dominant position in the global orthopedic implant market, mainly due to their superior mechanical resistance. However, their performance might be jeopardized due to the possible release of metallic debris, leading to cytotoxic effects and inflammatory responses in the body. Poly (ether-ether-ketone) (PEEK) is a biocompatible, high-performance polymer and one of the most prominent candidates to be used in manufacturing bone implants due to its similarity to the mechanical properties of bone. Unfortunately, the bioinert nature of PEEK culminates in its diminished osseointegration. Notwithstanding, PEEK’s bioactivity can be improved through surface modification techniques and by the development of bioactive composites. This paper overviews the advantages of using PEEK for manufacturing implants and addresses the most common strategies to improve the bioactivity of PEEK in order to promote enhanced biomechanical performance.

## 1. Introduction

Population aging, sports injuries, traffic accidents, and overweight are some of the many factors that lead to increasing demand for orthopedic implants provoking public health concerns [1]. For example, bone tissue loss is a common condition in elderly people. It causes an alteration in the microstructures of bone, reducing bone strength and density, which might eventually increase the predisposition to fractures [2]. In addition, osteoarthritis is a degenerative joint disease in which longer life expectancy and being overweight are some of the most prominent risk factors [3,4]. Furthermore, a more active lifestyle increases the risk of injuries. Indeed, orthopedic fractures are the most common injuries in running-involved sports and road traffic accidents [5]. Thus, the demand for bone substitutes increases globally, and the orthopedic market value is expected to expand progressively, reaching $79.5 billion by 2030 [6].

Bone is a dynamic tissue that undergoes a continuous remodeling process. However, spontaneous healing and repair may fail in the case of large bone defects or pathological fractures [7,8,9]. The use of metallic implants to tackle this issue is widespread, mainly due to their superior mechanical strength [10]. However, the harmful effects of the metallic implants, discussed in more detail further on, urge the use of alternative materials such as polymers and their composites. A promising implant material should be biocompatible, enable a good integration with the bone tissue, have a modulus closer to the bone to minimize bone resorption, and provide wear and corrosion resistance. Moreover, it should combine contradictory properties. For instance, the implant must be stiff and able to resist deformation. Nonetheless, it must also be flexible enough to absorb energy when deformed. Furthermore, it is expected to shorten and lengthen when compressed, as well as stretch and narrow when subjected to tension without cracking [9].

**Figure 1:**
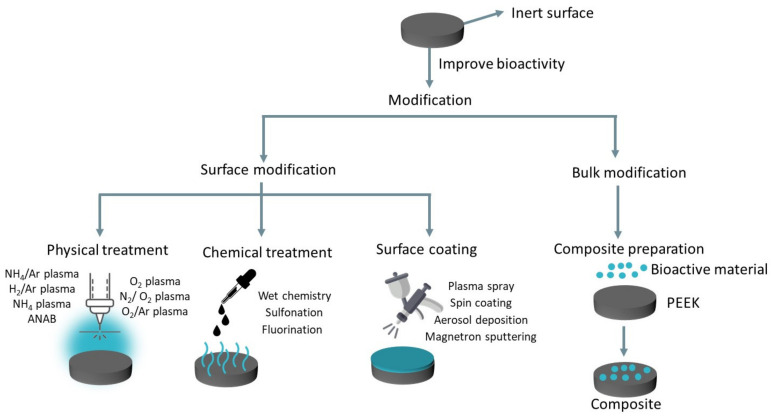
Techniques to improve the bioactivity of PEEK. Adapted with permission from [15]. Copyright 2014, MDPI.

Poly(ether-ether-ketone) (PEEK) was proposed as a biomaterial in 1998 by Invibio Ltd. (Thornton-Cleveleys, UK). Since then, PEEK-based materials have become an important group of biomaterials used in orthopedic and spinal implants owing to their outstanding properties [11]. Other clinical applications of PEEK include craniomaxillofacial reconstruction, dental implants, femoral stems, and total joint replacement. Studies have already shown that PEEK experiences fatigue resistance under dynamic load in simulated physiological conditions [12], good wear and corrosion resistance [13], and high creep resistance [14]. Notwithstanding, the hydrophobic nature of PEEK restricts protein and cell adhesion on its surface, hampering a good integration with bone tissue. Therefore, it is important to modify PEEK to enable it to promote both cell attachment and proliferation on its surface. It is well-established in the literature that some techniques allow the hydrophilicity of PEEK to be improved, consequently promoting osseointegration [15]. These techniques include physical and chemical treatments, surface coating, and bulk modification with bioactive materials, as schematically illustrated in Figure 1 [15]. On the other hand, if one considers the mechanical strength of PEEK for load-bearing applications, this property might be significantly ameliorated with reinforcing fillers.

This overview presents and discusses the reported strategies used to modify PEEK properties in order to mimic the biomechanical properties of bone. The content summarized herein aims to highlight future directions for manufacturing PEEK implants.

## 2. Drawbacks in Metallic Implant Devices

Over the past decades, metals have dominated the orthopedic implant market [16,17]. Nonetheless, postoperative observations have usually shown that the biomechanics of metallic implant devices requires improvement. Appropriate selection of the implant material is crucial for the long-term success of the orthopedic device. On this matter, using polymers and their composites is a promising solution since the final properties of the implanted material can be better tailored. PEEK is the leading high-performance thermoplastic candidate for replacing metal implant devices [18] and can tackle some drawbacks associated with the use of metallic implants.

**Figure 2:**
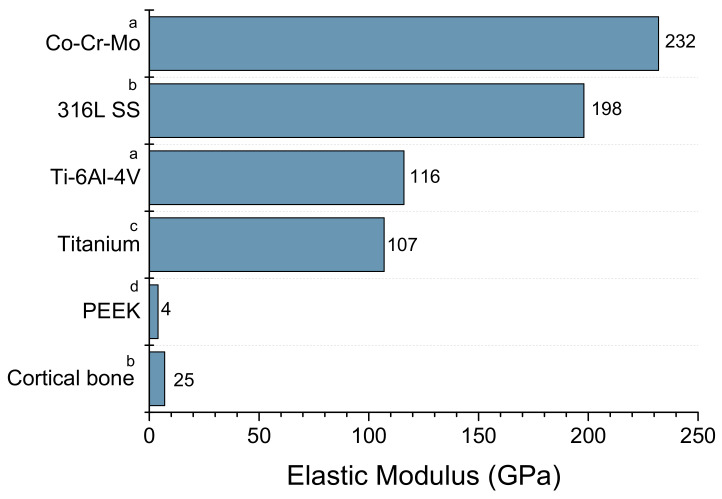
Elastic modulus of bone, PEEK, and metals usually used in implants (a) [22]; (b) [23]; (c) [24]; (d) [25].

**Figure 3:**
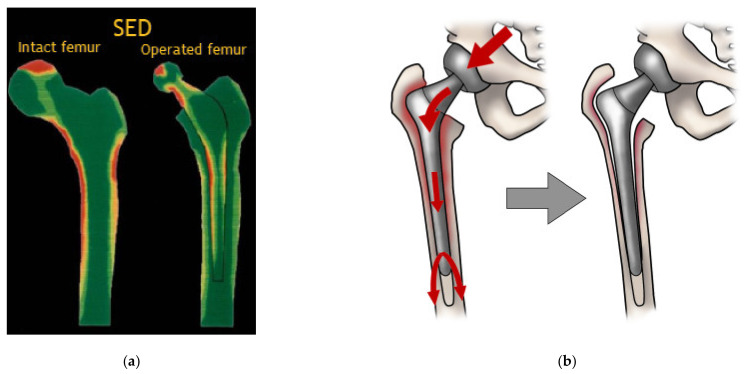
(**a**) Strain energy density in a healthy femur and on an operated femur. Reproduced with permission from [19]. Copyright 2015, Elsevier; (**b**) Schematic representation of stress shield and bone loss. Reproduced with permission from [26]. Copyright 2020, MDPI.

A common problem related to metallic implants in load-bearing applications is stress shielding. This phenomenon occurs because metals, such as cobalt-chromium-molybdenum alloy, 316L grade stainless steel, titanium-aluminum-vanadium alloy, and titanium, are much stiffer than the host bone, as shown in Figure 2. Consequently, the mechanical stimulus in the adjacent bone changes after implantation, and the physiological loading is mainly transferred to the implant [19]. Figure 3a shows the strain energy density (SED) of an intact femur (left) and the SED distribution immediately after placement of the implant (right). It is observed that high levels of SED in the femur (red) are greatly reduced following implant placement [19]. This reduction indicates that natural bone experiences decreased load stimulation compared to its natural state [20]. Over time, the lack of load stimulus weakens the bone, reducing its density. Consequently, bone loss surrounding the implant takes place, eventually leading to implant loosening and requiring revision surgery (Figure 3b) [20,21].

**Figure 4:**
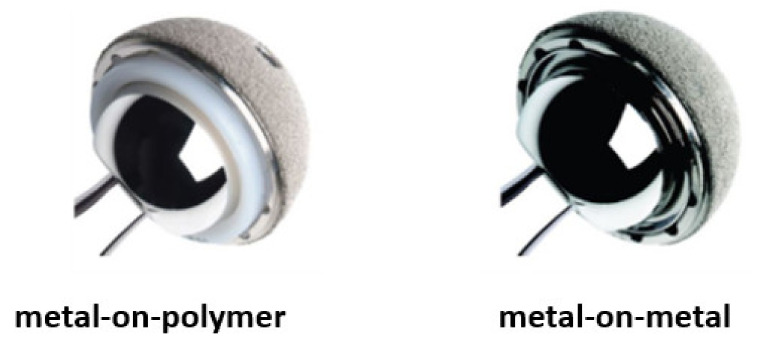
Materials components in hip implants. Reproduced with permission from [35]. Copyright 2003, Baishideng Publishing Group.

**Figure 5:**
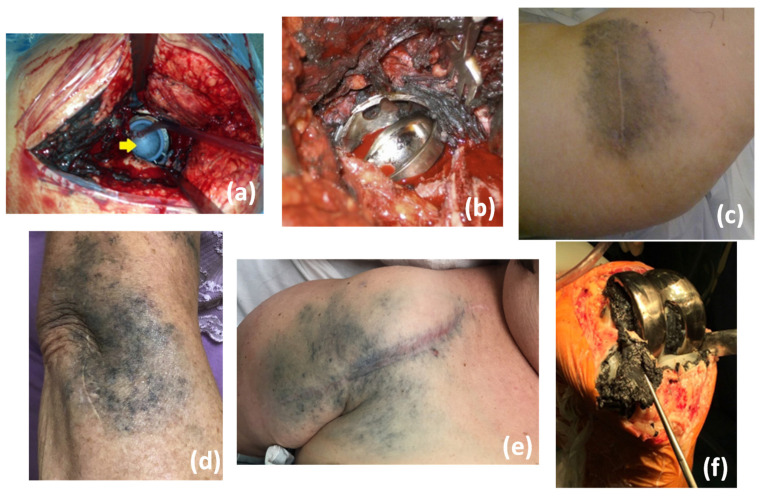
Metallosis in hip prosthesis with MoP (**a**) [36] and MoM (**b**) [37] implants; cutaneous manifestation of metallosis in the hip (**c**) [37], elbow (**d**) [38], shoulder (**e**) [29], and knee (**f**) [39] implants. Reproduced with permission from [29] Copyright 2018, Elsevier; [36] Copyright 2015, Elsevier; [37] Copyright 2012, Elsevier; [38] Copyright 2022, Elsevier; [39] Copyright 2020, Elsevier.

Another problem commonly observed in patients with metallic implants is metallosis, a medical condition characterized by the release of metallic wear debris into periprosthetic tissues and blood [27]. Metallosis causes a combination of direct cytotoxic effects, as well as an inflammatory response within the synovial and periarticular tissues, culminating in implant failure [28]. Metallosis is typical in joint prostheses, where the body movement induces friction between the implant components. It is known that metallosis can occur in both metal-on-metal (MoM) and metal-on-polymer (MoP) joint prostheses, illustrated in Figure 4 [29]. However, in the study by Lanting et al. [30], they demonstrated that the MoP hip prosthesis exhibited a negligible amount of material loss. In contrast, the MoM hip prosthesis had five times the amount of material loss, highlighting the superiority of polymeric parts in reducing metallic wear debris. Figure 5 shows a surgical revision performed in patients with MoP Figure 5a and MoM Figure 5b hip prosthesis in which the periprosthetic tissues showed necrosis and staining with metal debris. In Figure 5c, it is possible to note a cutaneous manifestation of metallosis caused by the MoM hip implant. This medical condition can also be observed in other joint replacements such as elbow, shoulder, and knee, as illustrated in Figure 5d–f. The release of metallic alloying debris after arthroplasty is a reason for concern. High serum cobalt levels, for example, can result in hearing and vision loss [31,32,33], and can even lead to death due to poisoning [34].

Metallic devices are prone to corrosion due to the harsh body fluid environment they are exposed to. In vivo corrosion resistance in implants is a key factor in assuring their functionality and biocompatibility. Depending on the level of corrosion, the biomaterial might lose its mechanical properties. Furthermore, cytotoxic and carcinogenic metal ions may be released during corrosion, triggering allergy, inflammation, and even metal poisoning [40,41]. Compared to metals, polymer materials have superior corrosion resistance against organic fluids and are already studied to be used as a coating in metallic materials [42,43,44]. Wei et al. [42] investigated the coating of AZ31 Mg alloy with poly-L-lactic acid, and the results showed that the polymer increased the corrosion resistance of the metallic material in a physiological environment.

**Figure 6:**
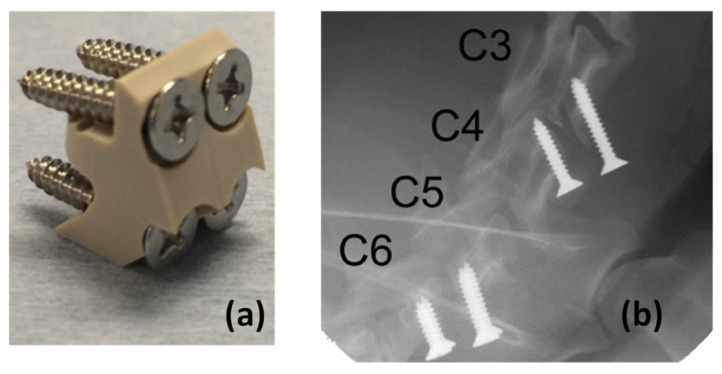
(**a**) Interbody spacer device and (**b**) postsurgical X-ray examination. Reproduced with permission from [48]. Copyright 2017, PLoS ONE.

Another advantage of polymer-based devices is their radiolucency, which allows for improved X-ray and computed tomography (CT) imaging compared to radiopaque metals [45]. In the case of X-ray, less intensive radiation is used, the image accuracy and definition are retained, and the patient’s exposure to radiation is reduced [46]. Figure 6 shows an interbody PEEK spacer implant fixed in adjacent vertebrae with stainless screws. As can be seen, PEEK is not detected in the X-ray image while the screws are observed. Radiolucency materials allow for examination of the bone underlying and surrounding the implant without occlusion or obstruction [47].

**Figure 7:**
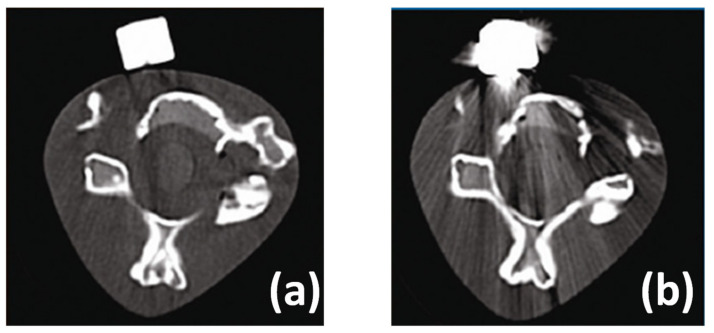
CT images (**a**) with a PEEK-OPTIMA image contrast grade and (**b**) with a metallic implant. Reproduced with permission from [46]. Copyrigth 2019, Elsevier.

Metallic implants are also known to create artifacts in magnetic resonance imaging (MRI), which may significantly hinder the ability of researchers and clinicians to visualize tissue proximal to the implant [47]. Figure 7a shows an implant made of PEEK from Invibio Ltd. [46] with a moderate ability to absorb X-rays, i.e., it is not completely transparent in the image. On the other hand, Figure 7b shows a metallic implant that is completely radiopaque. It is possible to observe that the metallic implant image contains artifacts and a “starburst” pattern radiating from the implant site [46]. A clinical study has already demonstrated that radiopaque implants help to detect local recurrence (cancer that has returned at or near the same location as the original tumor) due to the absence of artifacts [49].

Besides the aforementioned advantages of polymeric materials over conventional metallic orthopedic materials, PEEK devices are comparatively lightweight, offering ergonomic benefits to the patient. Furthermore, the high melting temperature of metals makes any melting processing step extremely energy-intensive and expensive. Table 1 compares density in metals, PEEK, and natural bone. It also shows the melting temperatures of the traditional materials used in orthopedic devices.

## 3. Peek Synthesis

**Figure 8:**
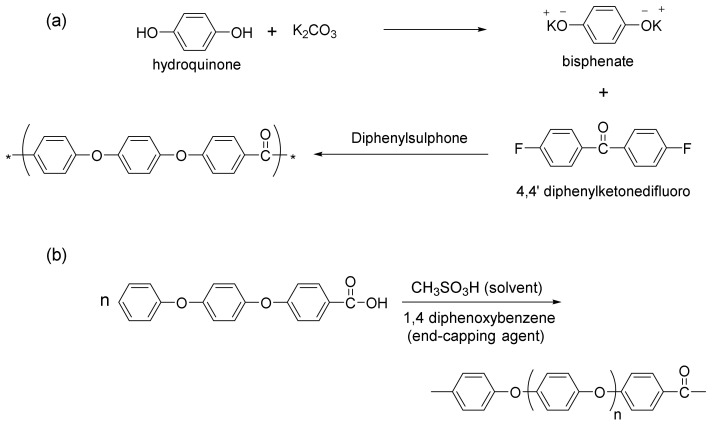
(**a**) Nucleophilic and (**b**) electrophilic PEEK synthesis. Adapted with permission from [53]. Copyright 2019, Elsevier.

Regarding the materials’ synthesis, there are two main PEEK polycondensation processes: the nucleophilic and the electrophilic routes. Most PEEK used in industrial applications is synthesized by the nucleophilic route patented in 1977 and commercialized by the brand Victrex PEEK [53]. This method involves a nucleophilic displacement reaction illustrated in Figure 8a. First, hydroquinone and sodium carbonate form bisphenate in situ and then react with a 4-4′difluorobenzophenone. Diphenylsulphone is the solvent, and the reaction is carried out at relatively high temperatures (>300 °C) [53,54,55]. By contrast, the electrophilic PEEK synthesis has limited commercial success since the produced materials have reactive-end groups, which are thermally unstable, such as benzoic acids [53]. Thus, due to its high thermal instability, the formed PEEK needs to be synthesized with an end-capping agent [56]. A modification in the electrophilic route has been proposed by Kemmish and Wilson [57], allowing the formation of a thermally stable PEEK that has been applied in industrial processes. Figure 8b summarizes this electrophilic process.

The manufactured PEEK is a rigid high-performance semicrystalline engineering thermoplastic. It is known for its outstanding thermal stability and high mechanical strength, as well as for its wear and chemical resistances. Besides, PEEK is radiolucency, bioinert, and has radiation stability. PEEK also has an average melting temperature of 343 °C and a glass transition temperature of 143 °C [56,58,59,60]. Table 2 displays some mechanical and thermal characteristics of PEEK.

## 4. Peek Bioactivity

**Figure 9:**
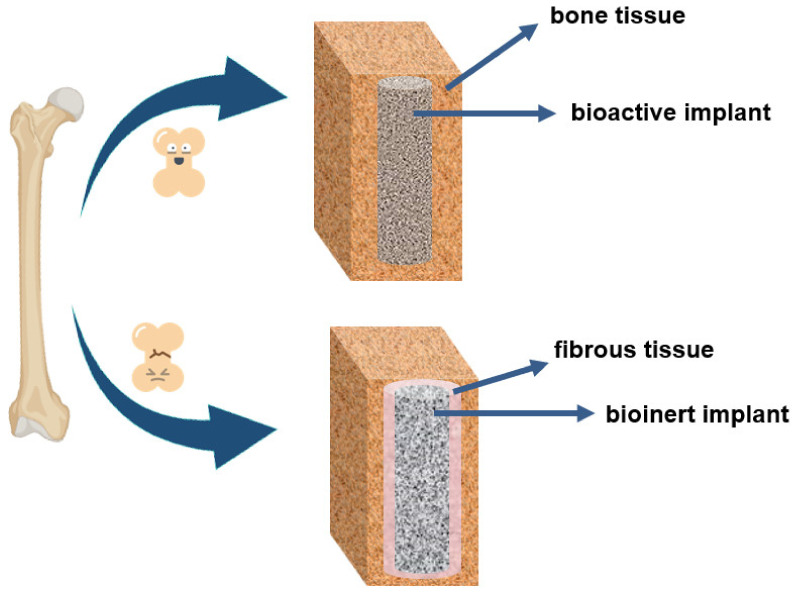
Body response to a bioactive and bioinert material.

As aforementioned, PEEK is a bioinert material with poor bonding to the surrounding tissues leading to unsatisfactory bone–implant integration. The lack of osseointegration along the implant–bone interface can lead to implant loosening due to its encapsulation by fibrous tissue and/or colonization by bacteria caused by the foreign body reaction that happens after the surgery, as schematically illustrated in Figure 9 [61,62,63].

**Figure 10:**
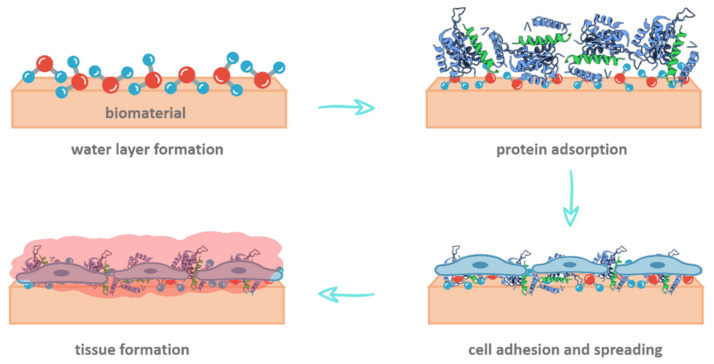
Schematic representation of the consecutive events on biomaterial surface subsequently to implantation [68].

When a biomaterial is placed in a biological environment, the first molecules that reach the implant surface are those of water. Following that, proteins interact with the biomaterial, and this contact is affected by the adsorbed water molecules. Subsequently, the adhesion of cells interacting with the adsorbed proteins takes place, influencing tissue growth as schematically shown in Figure 10 [64,65]. Fibronectin is one of the more influential proteins that mediate the biomaterial–cell interaction and is strongly adsorbed onto hydrophobic surfaces. However, this strong interaction produces a structural deformation of the protein, affecting its capacity to bind cells. On the other hand, when fibronectin is adsorbed into hydrophilic surfaces, the interaction is weaker, and the protein preserves its structure and cell-binding ability [66,67].

Two methods are proposed in the literature to overcome PEEK’s hydrophobicity and its lack of bioactivity: (i) the development of composites with bioactive fillers, and (ii) PEEK’s surface treatment. This second method can be divided into direct surface modification (physical and chemical treatments) and surface coating techniques (Figure 1) [65].

### 4.1. Surface Modification

After surgery, bone implants are directly in contact with bone tissue. Therefore, the biological properties of their surface are important for osseointegration. In this concern, the surface modification technique aims to alter the surface characteristics of PEEK without affecting its bulk properties [15].

#### 4.1.1. Chemical Modification

Chemical modifications introduce chemical groups into PEEK’s surface, creating a series of surface-functionalized PEEKs. It changes the surface chemical structure of PEEK to generate an environment with a favorable cellular response [15,69]. Zheng et al. [70] prepared a series of modified PEEK with the functional groups -COOH, -OH, and -PO_4_H_2_. These groups were studied because they are capable of inducing apatite layer growth on their surface in the presence of simulated body fluid. The subsequent tests showed that these species presented beneficial properties supporting cell adhesion, spreading, proliferation, and higher osseointegration compared to pure PEEK. Sulfonation of the PEEK chain is another treatment that improves the polymer hydrophilicity and bioactivity [71]. Concentrated sulfuric acid is the most common sulfonating agent and produces a porous 3D network on the PEEK surface. Ouyang et al. [71] proposed a hydrothermal treatment to remove the residues on the surface. The thermally treated samples showed better osseointegration and antibacterial ability when compared to the untreated sulfonated PEEK. Another way to introduce sulfonate groups into PEEK’s surface is the treatment with the so-called “piranha” solution. Dos Santos et al. [69] compared the sulfonation process with sulfuric acid and piranha solutions and proved that both methods were efficient in supporting fibroblast adhesion and proliferation. A further chemical modification is the amination of PEEK. The addition of amine groups into PEEK is a powerful method to promote bioactivity since it serves as a base for the covalent immobilization of the cell-adhesive protein fibronectin. Liu et al. [72] introduced amino groups into PEEK using (3-aminopropyl) tri-ethoxy silane as an amination agent. The study showed enhanced hydrophilicity and fibronectin adsorption on the aminated PEEK. This improvement was subsequently in vivo manifested as better tissue integration. Chen M. et al. [73] combined a physical and wet chemical treatment to produce a fluorinated PEEK (PEEK-F). Argon plasma immersion ion implantation was employed, followed by hydrofluoric acid treatment. PEEK-F showed increased cell adhesion, spreading, and proliferation, and better osseointegration was achieved than in pure PEEK. Another combination of physical and chemical methods was used to graft phosphonate groups into PEEK. For this purpose, sandblasting and two-step diazonium chemistry treatments were used. The in vivo test showed that after three months of implantation, the untreated PEEK implant was surrounded by fibrous tissue. Nonetheless, in the treated PEEK, apatite mineral deposition was observed in the region between the treated implant and the underlying bone [60].

#### 4.1.2. Physical Modification

The commonly used physical treatments to modify PEEK’s surface are plasma, laser, accelerated neutral atom beam (ANAB), and ultraviolet (UV) irradiation. The plasma treatment was used to alter the surface chemistry of the material. Nitrogen [74,75], oxygen, argon [76], water [76,77], ammonia [77], and air [78] are some plasma sources that introduce functional groups into the PEEK surface. These introduced polar groups increase the surface hydrophilicity and roughness, conditions for positive cell interaction. An in vitro study was performed in PEEK treated with a gas mixture of water vapor as a plasma resource and argon as an ionization assistant [76]. The modified polymer exhibited a more favorable environment for osteoblast adhesion, spreading, proliferation, and early osteogenic differentiation. Therefore, it is expected that a faster bone maturation induction will occur around the PEEK implant [76]. Laser treatment is a low-cost technique that increases the material’s surface energy, increasing the surface roughness and wettability [79]. Similar to plasma treatment, laser technology allows for the addition of polar groups on the polymer surface, increasing the potential for cell adhesion and thus increasing the likelihood of implant acceptance by the body [80]. Zheng et al. [81] proposed a dual modification method that combines laser and plasma surface treatments. While the laser treatment constructs microstructures over the PEEK surface, the plasma polymerization of acrylic acid introduces carboxyl groups onto the PEEK surface. The dual-modified PEEK was more favorable for pre-osteoblast adhesion, spreading, and proliferation. Plasma and laser techniques can also be used to immobilize biomolecules on the PEEK surface [74,82,83,84,85,86]. Terpiłowski et al. [74] pre-treated PEEK with nitrogen plasma to further immobilize chitosan on its surface. Chitosan exhibits an intrinsic antibacterial activity and is an alternative to be introduced in implant materials to avoid the bacterial resistance provoked by the excessive use of antibiotics. It was observed that the plasma activation of PEEK increased the adhesion of chitosan to its surface due to a combination of two factors. The increased surface roughness, along with the interaction of the amine groups on chitosan and the nitrogen deposited on the surface, increased chitosan linkage to the polymer. Gelatin is a protein derived from collagen. It is the major protein in the extracellular matrix and has been widely studied due to its outstanding biocompatibility and cost-effectiveness [87,88]. Omrani et al. [85] performed a pre-plasma treatment on PEEK’s surface to enhance the affinity between gelatin and PEEK. They found that the immobilization of gelatin into PEEK promoted higher cell growth than both plasma-treated and pure PEEK. The ANAB process does not change the PEEK chemical structure but modifies its hydrophilicity due to changes in surface roughness. Khoury et al. [89] demonstrated that after ANAB treatment, PEEK showed an increase in cellular adhesion and proliferation activity. Finally, UV irradiation was performed as the first stage of some chemical modification by introducing active functional groups into the bioinert PEEK surface. It happens through the reaction of radical species generated by the diphenyl ketone structure present in PEEK when exposed to irradiation with a monomer. Sulfonate [90] and phosphate [91] chemical groups were introduced to PEEK by this technique, producing a surface group-functionalized PEEK. Both modified PEEK greatly enhanced the adhesion, spreading, proliferation, and osteogenic differentiation of the pre-osteoblastic cells after surface sulfonation and phosphorylation.

#### 4.1.3. Limitations of Chemical and Physical Treatments

Some drawbacks in the treatments reported above lie in the recovery of the initial PEEK hydrophobicity [76,92]. This aging phenomenon can be attributed to the reorientation of polar groups within the polymer matrix to reduce their surface energy, reverting the modified PEEK to a surface close to its original property [92,93]. For example, the physical treatment with plasma has already been demonstrated to revert PEEK to its original surface in a few hours or days, while in the chemical treatments, the aging can be retarded for tens of days [92]. Furthermore, grafting polar groups on the PEEK surface may also result in an unstable surface once the bonding may not tolerate the sterilization process crucial in biomedical applications [94]. Wang et al. [76] studied the aging process after subjecting PEEK to a plasma treatment using a mixture of water and argon. For that study, the produced samples were stored either in air or in water, followed by air. A decrease in the contact angle was observed right after the plasma treatment. However, the contact angle increased in samples stored in the air after only four days, reaching a value higher than the one found for pure PEEK. On the other hand, the samples stored in water or water followed by air, after 15 days, displayed relatively stable contact angle values. Since the implants are stored for a substantial amount of time before implantation [94], hydrophobic recovery is of concern, and the storage of the implants in water is not a solution able to be applied in the industry.

#### 4.1.4. Surface Coating

The deposition of a bioactive layer on PEEK’s surface is another modification process to improve its cell affinity. Several techniques are feasible for applying bioactive coatings, such as electron beam evaporation, arc ion plating, plasma spraying, plasma immersion ion implantation, chemical deposition, and spin coating. The deposition of bioactive materials such as titanium dioxide (TiO_2_) [95] and calcium metasilicate (CaSiO_3_) [96] by e-beam was reported in the past decade. Both studies revealed that the coated substrates presented a better bone–implant contact than the pure PEEK. TiO_2_ can also be coated into PEEK through the arc ion implanting process. The TiO_2_-PEEK substrate studied by Tsou et al. [97] showed a bone-bonding performance superior to the pure PEEK. The coating with titanium (Ti) by the plasma-sprayed technique was already studied by Walsh et al. [98]. After the deposition, a rough surface was formed, and the in vivo study demonstrated that direct bone–implant bonding was achieved by Ti-PEEK substrate.

Furthermore, the stiffness at the bone–implant interface with Ti-bond was significantly greater than in uncoated PEEK. Tantalum (Ta) [99] and calcium (Ca) [100] can also be coated into PEEK using plasma immersion ion implantation, producing a material with better cell adhesion and proliferation and enhanced osteogenic activity. Moreover, the Ta-PEEK sample presented an elastic modulus closer to that of the human cortical bone than the uncoated PEEK. Hydroxyapatite (HA) is one of the most common bioactive materials used for coating biomaterials. Almasi et al. [101] coated HA into PEEK by chemical deposition. In this technique, PEEK was first sulfonated, introducing -SO_3_H groups, responsible for the increase in the surface roughness, and then HA was deposited. The obtained material presented an increase in wettability, which is an indication of improved bioactivity. The deposition of HA through the spin-coating technique was also reported by Johansson et al. [102]. Their results of the in vivo experiments showed that a higher bone area was formed surrounding the HA-PEEK implant compared to the untreated PEEK.

As evidenced, the bioinertness of PEEK can be greatly enhanced by means of the coating of bioactive materials. Nonetheless, some difficulties are associated with this method. For example, coating PEEK with metallic materials may trigger problems already associated with the use of metallic implants. For instance, stress shielding, due to an increase in the elastic modulus and the release of metal ions, can increase the risk of inflammation and implant loosening [103,104]. Furthermore, since wear or delamination may be caused by shear loading, Kienle et al. [104] carried out a mechanical test to investigate whether the impaction process of Ti-coated PEEK can trigger one of these phenomena. The results showed the loss of some coating materials in the plasma-sprayed Ti implants, although full delamination was not observed.

It is worth mentioning that even thin coatings can interfere with the clinical analysis of the bone–implant interface owing to artifacts produced during medical imaging techniques [105]. Among the described methods, the deposition of HA via plasma spraying is a method qualified for commercial operation [65]. However, this technique is expensive and cannot be easily applied to PEEK implants with complex shapes [60]. In addition, the difference in stiffness between the substrate and the coating can aggravate the stress at the interface, leading to the delamination of the coating [106]. Moreover, HA coatings suffer from insufficient adhesion to PEEK due to a low bonding strength between the PEEK and the HA layer, which can also produce debonding at the interface [60,107].

### 4.2. Bulk Modification

An alternative to overcome the aforementioned surface modification shortcomings is to tailor PEEK’s properties by compounding it with nanoparticles. The melt-processing temperature of PEEK ranges between 360 and 400 °C, and it can be processed using all of the typical thermoplastic processes, such as injection molding, extrusion, and compression molding. The high processing temperature and inertness in most solvents hinder PEEK processability, making it a challenging procedure. In contrast, its high thermal and chemical stability provides remarkable resistance to sterilization by gamma and electron beam radiation, an important attribute in biomedical applications, among other advantages [56].

#### 4.2.1. Bioactive Composites of PEEK

Different bioactive materials such as TiO_2_ [108], bioglass [109], calcium silicate [110], β-tricalcium phosphate [111,112], natural amorphous silica fibers [113], HA, and HA doped with fluorine [114], as well HA doped with strontium (Sr) [115], have already been incorporated to PEEK to mitigate its bioinertness (Table 3). Among these bioactive fillers, the incorporation of HA to PEEK draws much attention and is extensively studied [15,58,116,117,118,119,120,121]. HA is the major inorganic bone component, and it is known for its biocompatibility, bioactivity, and osteoconduction properties [15]. Therefore, this section will focus on the mechanical and biological properties of PEEK/HA composites.

**Figure 11:**
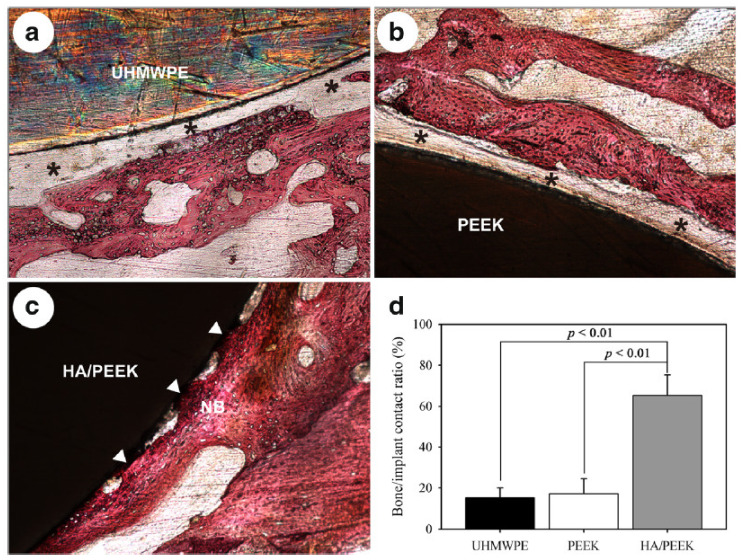
Histological observation after eight weeks of implantation in rabbits: (**a**) Ultra-high molecular weight polyethylene (UHMWPE), (**b**) PEEK, (**c**) HA/PEEK, (**d**) quantitative analysis of bone/implant contact ratio. The black asterisks indicate the fibrous connective tissue, and the white arrows indicate the bone contact. Printed with the permission of [116]. Copyright 2019, BioMed Central.

One way to compound PEEK and HA is by using ball milling and injection molding processes, as reported by Ma and Guo [116]. The tensile test revealed that the elastic modulus increased as the HA content increased (from 0 to 40 wt%). The addition of 30 and 40 wt% of HA provided an elastic modulus of approximately 7.2 and 10.6 GPa, respectively, while the value for the pure PEEK is only 2 GPa. Since the elastic modulus for cortical bone ranges from 7 to 25 GPa, the prepared composites match the bone stiffness. On the other hand, the tensile strength decreased with increasing HA content, indicating a loss of ductility. The composite with 30 wt% of HA was selected to study its bioactivity since both elastic modulus and tensile strength match the values of these properties for bone (Table 4). As expected, the PEEK/HA composite presented a higher cell attachment, proliferation, and osteogenic activity than pure PEEK. In fact, the hydrophobic surface of pure PEEK hinders cell attachment, which leads to its separation from the bone. Both samples were immersed in SBF to evaluate the bioactivity of PEEK/HA and PEEK. It was observed that the composite induced apatite formation after seven days of immersion. After 28 days, the composite was almost completely covered by apatite, while no changes were observed on the pure PEEK surface. In addition, the in vivo test showed that after eight weeks of implantation, new bone was formed and integrated with the implant surface of PEEK/HA. However, the pure PEEK surface was surrounded by fibrous connective tissue (Figure 11) [116].

Ma et al. [117] proposed an in situ synthesis process to produce PEEK/HA composites in order to improve the interfacial bonding between PEEK and HA and overcome the high-temperature processing issue. Di-terbutyl peroxide, p-dihydroxybenzene, sulfobenzide, K_2_CO_3_, and Na_2_CO_3_ were used for PEEK synthesis, and different contents of commercial HA powders were introduced to the reaction medium. Lower HA contents (2.6 and 5.6 vol%) increased the tensile strength of the composites. The composite reached the higher tensile strength of 106 MPa for 2.6 vol% of HA. However, higher HA contents decreased the strength, probably due to filler agglomeration. Despite the decrease in the tensile strength, the composite with 8.7 vol% of HA displayed a tensile strength of 75 MPa, which is in the range of the tensile strength of the cortical bone (50–150 MPa) (Table 4). Although the elastic modulus of the composites was not reported, an increase in this property is expected with HA increment. A subsequent study by Ma et al. [122] evaluated the in vivo biocompatibility and toxicity of the composite with 5.6 vol% of HA and the in vivo bioactivity of all composites [122]. The results showed that the PEEK/5.6 vol% HA composite has desirable biocompatibility without apparent toxicity to animals. Moreover, the bioactivity test demonstrated that a higher HA content promotes faster new bone tissue growth around the implant made of PEEK/HA. Unfortunately, this method is not suitable to be used on an industrial scale due to the complex preparation process.

A current proposal by Zhong et al. [118] to produce PEEK/HA composites is the three-dimensional (3D) printing of a HA scaffold. Then, the incorporation of PEEK into the scaffold uses the compression molding process, finally soaking the composite in an HCl solution to introduce porosity. The HCl solution dissolves the HA network, leaving interconnected channels within the composite. The composite, containing 40 vol% of HA, presented good biocompatibility, and the compressive strength (110 ± 7 MPa) is in the range of the cortical bone (100–230 MPa).

**Figure 12:**
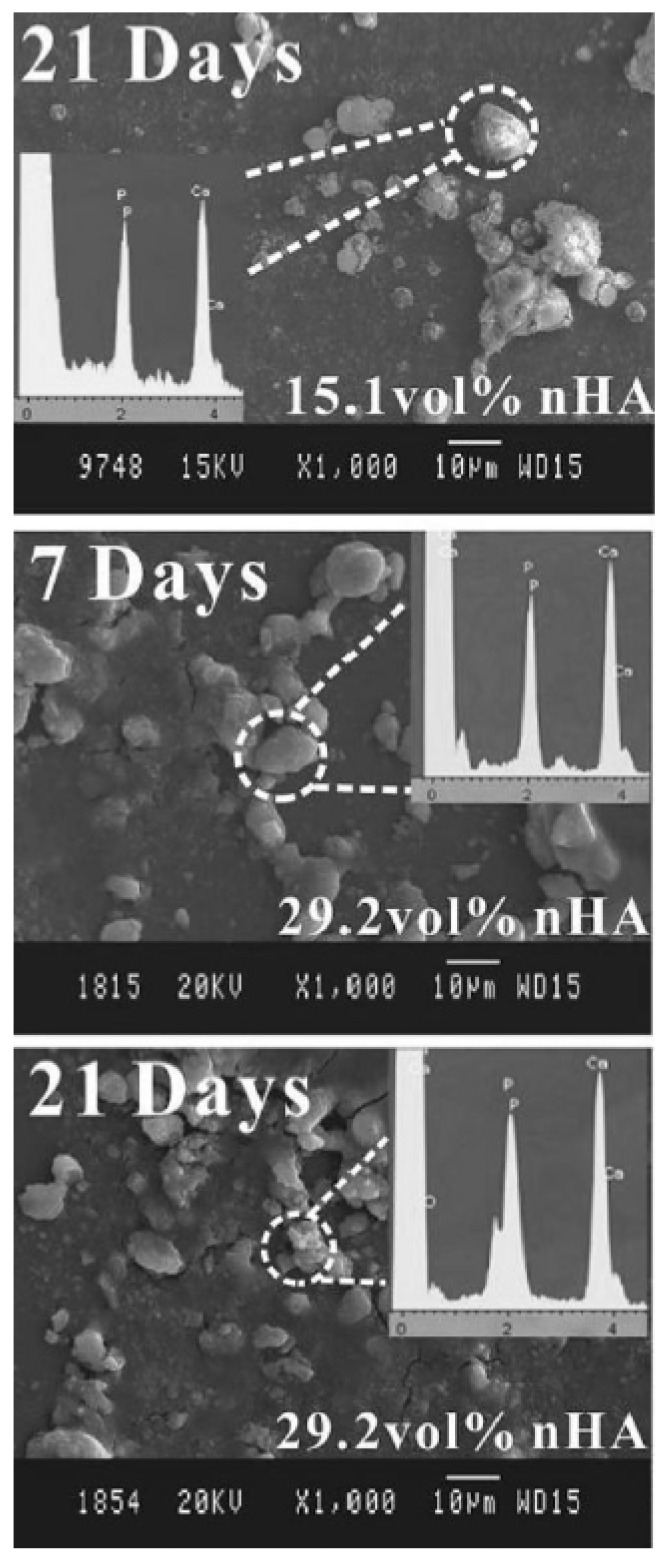
SEM micrographs after immersion in SBF on the 15.1 vol% nHA/PEEK and 29.2 vol% nHA/PEEK nanocomposites. The deposition of the apatite mineral layer is observed in the images and confirmed by energy-dispersive X-ray spectroscopy. Printed with the permission of [58]. Copyright 2012, Wiley.

Another way to prepare these composites is first to disperse PEEK and HA in a solvent to prevent the agglomeration of HA particles in the PEEK matrix. In Li et al.’s [58] study, PEEK and nanorod HA (nHA) contents were independently dispersed in ethanol under sonication. The separate suspensions were then mixed and kept under continuous magnetic stirring. The mixtures were dried and cold-compressed. In addition, the samples were sintered under a protective argon atmosphere and cooled at room temperature. In conjunction with a high filler addition, the high melt temperature of PEEK culminates in large melt viscosity and poor processability of the composites. For this reason, Li et al. [58] implemented the aforementioned methodology to avoid conventional techniques such as injection and extrusion. From mechanical tests, they observed, like in the other studies, that the tensile strength decreases with increasing filler content (15.1 to 38.2% vol nHA). Using nHA can potentialize this drop in strength, since nanofillers tend to aggregate when their contents become high due to their large surface area. On the other hand, the elastic modulus increases with increasing nHA content (Table 4). The mechanical properties of the composites filled with 21.6 and 29.2 vol% closely matched those of human cortical bone (Table 3). The in vitro bioactivity test performed in the composites with 15.1 and 21.9 vol% nHA demonstrated that both materials are bioactive. However, fewer apatite minerals were deposited on the PEEK/15.1 vol% nHA surface, as shown in Figure 12. The cytotoxicity test confirmed that higher nHA content induces higher biocompatibility. 

An attempt to improve PEEK-HA bonding is to modify the HA surface. Ma et al. [119] reported a silanization of HA. In their work, the HA was modified with a silane coupling agent (KH560), and the modified HA (mHA), as well as HA without modification, was mixed with PEEK powder at different ratios (5 to 30 wt% of HA or mHA). Finally, the mixtures were hot-pressed at 320 °C. The PEEK/mHA composites presented a higher elastic modulus than the unmodified PEEK/HA composites (Table 4). The tensile strength increased for low HA and mHA contents (5 wt%) and then decreased with the increment of filler content. Comparatively, the tensile strength was higher for the PEEK/mHA composites, showing that the modification on the HA surface provided a better bonding between the filler and the polymer matrix. The in vivo analysis revealed that new bone layers around the implants with the modified filler were larger than in the pure PEEK. Moreover, the growth of bone tissue around the PEEK/5 wt% mHA was the highest among all composites [119]. The better results of the composite with 5 wt% mHA were attributed to an efficient dispersion of the inorganic filler in the organic matrix. Regarding the content of silane coupling in the filler, the thermogravimetric analysis indicated that the coupling agent covered only 1.3 wt% of the total quantity of HA. Thus, the composite’s mechanical properties should be greatly improved with the modified filler to justify this additional cost in production.

As seen in the reviewed studies [58,116,117,118,119], as well as in other works reported in the literature [120,121], adding large amounts of HA to PEEK increases the elastic modulus; however, it turns the material more brittle. On the other hand, the increase in the HA contents generally improves the biological properties of the material. Therefore, it is a challenge to combine good biological and mechanical properties. Aiming to use this material in load-bearing applications, it is important to produce a resistant material since the implant will be continuously loaded at relatively high stress levels. Nevertheless, the implant must be bioactive to provide a good biological fixation, avoiding failure. The poor interfacial bond between PEEK and HA is responsible for the ineffective load transfer across the filler–matrix interface, leading to the debonding of these materials [58]. This interfacial debonding contributes to the initiation and propagation of micro-cracks, which can cause fatigue failure [121]. As recognized, coupling agents are widely used to facilitate stress transfer across the filler–polymer. However, this agent might cause cytotoxicity during biological tests. In addition, due to the high melting temperature of PEEK, it is difficult to find a coupling agent that can withstand high temperatures without releasing volatiles. Therefore, finding a balance between mechanical strength and biological properties is important. This balance can be accomplished by reinforcing PEEK with an appropriate filler, as discussed in the following section.

#### 4.2.2. Reinforced PEEK Composites

One of the first fillers added to PEEK to improve its stiffness and strength was carbon fiber reinforcement (CFR). For instance, PEEK with CFR has an elastic modulus in the range of the cortical bone, i.e., around 20 GPa (Table 4), while the elastic modulus for pure PEEK is 4 GPa [123]. On the other hand, the presence of CFR impairs the strain properties of the material, as indicated by Kurtz and Devine, 2007 [56]. Over the last years, PEEK with CFR has been introduced in spinal and orthopedic implants [11,124].

The biofunctionalization of PEEK is recommended to enhance its bioactivity. For example, in an attempt to obtain a composite with proper biomechanics, HA was added to the PEEK/CFR composite [125]. The presence of CFR in the ternary PEEK/CFR/HA composite enhanced the strength loss derived from the addition of HA into PEEK. Deng et al. [125] prepared PEEK/25 wt% nHA/20 wt% CFR by melt blending and injection molding. The ternary composite showed an elastic modulus of 16.5 ± 0.07 GPa, which was higher than the values usually found for the binary PEEK/HA composite. These values usually reach the lower range of the elastic modulus of cortical bone (7–20 GPa). According to Deng et al. [125], the tensile strength of the composite was higher than that of pure PEEK, unlike what was observed in PEEK/HA composites, revised in the previous section. The subsequent in vitro tests showed that PEEK/nHA/CFR has better osteogenic differentiation, bioactivity, higher cell attachment, and proliferation. In addition, the in vivo evaluation revealed that the newly formed bone volume of the ternary composite was higher than that using pure PEEK. Moreover, Tan et al. [126] showed that PEEK/nano-HA/short carbon fiber bioactive composite provides the most suitable implant for bone plating application for tibia. Furthermore, Zhou and Yang [127] used carbon/PEEK composite plates and demonstrated that these lower-stiffness bone plates had reduced stress shielding at the fracture site.

Despite the improved mechanical properties promoted by the CFR, clinical concerns have already been reported in the literature regarding composites with CFR for biomedical applications. For example, a clinical study reported the failure of the PEEK/CFR tibial intramedullary nail 10 weeks after its placement [128]. Another clinical study showed that after wrist-plate implantation made of PEEK/CFR, the fibers became exposed directly to the living tissue, resulting in severe synovitis, which led to flexor tendon damage in the wrist [129]. The symptoms of an inflammatory response started after three months of the operation, where painful swelling was reported. The symptoms kept worsening, and after four months, the patient was unable to flex the thumb [129]. Therefore, it is important to better understand the toxic response of the CFRs in the human body, since other studies have already shown that carbon particulate debris is released in the tissue surrounding the PEEK/CFR implants [56,130].

The incorporation of zinc oxide (ZnO) and TiO_2_ into PEEK can simultaneously promote reinforcement and antibacterial activity in the matrix [131,132,133]. To date, both ZnO and TiO_2_ are generally recognized as safe and effective components by the Food and Drug Administration (FDA) [134].

Díez-Pascual, Xu, and Luque incorporated [132] ZnO and silanization ZnO (s-ZnO) to PEEK using a ball mill at cryogenic temperature, followed by compressing molding at 360 °C. The elastic modulus and tensile strength of the composite were higher for the s-ZnO than for the composite with unmodified ZnO. This behavior is ascribed to the strong reinforcement driven by the coupling agent, promoting better adhesion between the matrix and the filler. On the other hand, the elongation at break decreases with the increase in the ZnO and s-ZnO particles. This decrease in ductility was more pronounced for the composites with unmodified ZnO. The antibacterial activity tested against human pathogenic bacteria was improved by increasing the amount of ZnO and s-ZnO in the polymeric matrix [132]. However, a better antibacterial property was obtained for the PEEK/s-ZnO composites, and the best activity was achieved with 7.5 wt% s-ZnO. In a similar study, Hao et al. [135] modified ZnO with a different silane coupling agent in a similar study. However, the ZnO nanoparticles were processed with PEEK by a twin-screw extruder followed by injection molding. The tensile strength and the elastic modulus were improved after adding s-ZnO. However, the increase in mechanical properties with 5 wt% s-ZnO in this study was lower than the one observed in the PEEK/5 wt% s-ZnO composite developed by Díez-Pascual, Xu, and Luque [132]. Given the increase in the tensile strength and the elastic modulus for the PEEK/s-ZnO composites, the melting of the silane component during the compound process can lead to its decomposition due to the high melting temperature of PEEK. If the decomposition triggers the release of toxic volatiles, it might be of concern for biomedical applications. In another study, Díez-Pascual and Díez-Vicente [133] prepared a masterbatch of carboxylated PEEK and ZnO (PEEK-CO-O-CH_2_-ZnO), which was subsequently compounded with PEEK in a mini-extruder at 380 °C. The produced PEEK/masterbatch composites showed a higher stiffness, strength, and ductility than both the pure PEEK and the PEEK/ZnO composites prepared for comparison. The antimicrobial behavior of PEEK/masterbatch composites was similar to the one observed in their previous study, i.e., the antimicrobial effect increased by raising the amount of the nanoparticles and was found to be higher for PEEK/masterbatch composites. Montaño-Machado et al. [136] prepared PEEK composites with ZnO by extrusion. However, the amount of filler incorporated into the matrix was below the theoretical values and proved the adversity in introducing nanoparticles into the PEEK matrix due to its high melt viscosity, which also hinders a reasonable dispersion of the fillers. The preparation of PEEK/TiO_2_ composites using a single screw extruder was performed by Bragaglia et al. [137]. The presence of TiO_2_ slightly increased the stiffness of the material and barely affected the elongation at break and the tensile strength, not turning the material brittle. Although the antibacterial activity was not tested in the aforementioned study [137], in a previous study performed by Díez-Pascual and Díez-Vicente, [131] it was confirmed that the presence of TiO_2_ in the blend PEEK/PEI promoted antibacterial activity in the material.

Table 5 summarizes the notable finds of some composites of PEEK with different nanoparticles for biomedical applications.

### 4.3. PEEK on Biomedical Field: Applications and Future Prospectives

In the orthopedic segment, a commercial femoral stem containing PEEK in its current architecture is the VerSys^®^EPOCH^®^ (Zimmer, Warsaw, IN, USA) [138]. It is a composite of PEEK and Co-Cr-Mo alloy core coated with Ti fiber. Unfortunately, after EPOCH implantation, a clinical study reported implant failure due to the delamination of PEEK from the Co-Cr-Mo alloy core [139,140]. In dental implants, PEEK is used to construct partial dentures, crowns, and bridges. Spinal implants of PEEK are already on the market, like the ANATOMIC PEEK™ PTC (Medtronic, Fridley, MN, USA), [141] which is a cage for cervical fusion coated with Ti. Although the metallic coating can improve the wettability of the material generating better cell attachment, the delamination of Ti coating can cause the implant loosening. Additionally, wear debris of Ti can induce inflammation [104]. In the past few years, the subtractive manufacturing method has been the standard technique for manufacturing PEEK parts [142]. However, it displays shortcomings such as waste generation and no specific match with patient anatomy.

The growing need for implant customization leads to additive manufacturing (AM) as an emerging technique to fulfill this demand. This technology has attracted the attention of surgeons and patients, and its application is growing steadily. AM is a process used to make 3D-printed objects from a digital model by depositing successive layers of the material [142]. Despite the wide range of AM processes, fused deposition modeling (FDM) is a widespread technique for the AM of polymers, which permits the fabrication of complicated geometrical parts. In the FDM technique, layers are printed starting from polymeric filaments. During 3D printing, the filament is melted and extruded in a nozzle. Then, layer by layer, the material is deposited onto a heated building platform, following a specific laydown pattern described by the digital model [143,144]. Customized PEEK implants are already used for craniomaxillofacial reconstruction and can improve postoperative outcomes. During the surgery, if a prefabricated implant is used, it has to be fitted manually, increasing operative time and, consequently, the risk of contamination. Furthermore, a good esthetic appearance may not be achieved [145]. The increasing need for patient-specific implants to fit individual anatomical shapes shows the importance of 3D printing in the production of medical implants and opens the need for further investigations on the subject.

## 5. Final Considerations

The clinical interest in developing a material with adequate biomechanics is well recognized. PEEK has drawn considerable attention to being used in load-bearing biomedical applications. As extensively discussed in this work, its bioactivity should be enhanced. The surface modification techniques usually used for these purposes face more drawbacks than bulk modification. The main problem of the chemical and physical treatments relies on the recovery of the hydrophobicity of PEEK, jeopardizing the cell’s interaction. Furthermore, the grafting of polar groups commonly used may create an unstable surface. Moreover, the delamination that a surface coating material may undergo inefficient stress transference between the bone and the implant. Therefore, bulk modification emerges as a better route to enhance PEEK’s bioactivity. As evidenced, the addition of HA to PEEK, widely studied in the literature, turns the material brittle but improves its bioactivity. On the other hand, adding reinforcing fillers, such as CFR, ZnO, and TiO_2_, can significantly increase the mechanical strength of PEEK composites. However, the reinforced composite lacks bioactivity, a property that is a key factor in providing direct bone–implant bonding, avoiding implant failure. Therefore, it is still a great challenge to produce a PEEK-based material that can be used in load-bearing conditions. This overview intends to point out the drawbacks of using metals in implant devices and highlight that it is important to concentrate efforts on producing a tough and bioactive non-metallic material in an attempt to improve the quality of life of people all around the world.

## Figures and Tables

**Table 1:** Density and melting temperature of bone, PEEK, and metal alloys traditionally used in orthopedic implants.

Material	Density (g/cm^3^)	Melting Temperature (°C)	Reference
Cortical bone	1.5–2	-	[23]
Trabecular bone	0.2–0.6	-	[23]
PEEK	1.4	304–391	[25]
316L stainless steel	7.99	1380	[23,50]
Co-Cr-Mo	8.3	1350–1430	[51]
Ti-6Al-4V	4.43	1655	[52]

**Table 2:** PEEK mechanical and thermal properties.

Property	Value	Reference
Elastic modulus	2.0–4.0 GPa	[25,59]
Tensile strength	84 MPa	[59]
Compressive strength	112 MPa	[59]
Elongation at break	3.5%	[58]
T_g_	143	[56]
T_c_	314	[58]
T_m_	343	[58]

**Table 3:** Bioactive composites of PEEK and their processing method.

Filler	Processing Method	Bioactivity Highlight	Reference
nano-TiO_2_	Dispersion in ethanol and compression molding	In vivo studies showed that the percent of bone volume on the n-TiO_2_/PEEK surface was approximately twice as large as that of PEEK.	[108]
nano-bioglass	particle leaching and compression molding	The apatite mineralization ability in simulated body fluid (SBF) was significantly improved in the composite.	[109]
nano-calcium silicate	High-speed ball mill and injection molding	In vivo tests revealed that the composite promoted osseointegration at the bone/implant interface compared to PEEK.	[110]
β-tricalcium phosphate	laser sintering	In vivo evaluation showed that the composite exhibited bone–implant contact while the control group was encapsulated by fibrous tissue.	[112]
natural amorphous silica fibers	Ball mill and compression molding	The addition of fibers into PEEK stimulated the metabolic activity of fibroblasts grown on the composites compared to the metabolic activity of neat PEEK.	[113]
HA doped with fluorine	Dispersion in alcohol and compression molding	The composite exhibited enhanced antibacterial activity and osseointegration.	[114]
HA doped with Sr	Solvent dispersion and compression molding	The addition of the filler enhanced the bioactivity of the material.	[115]

**Table 4:** Mechanical properties of PEEK composites and cortical bone.

Material	Processing Method	Elastic Modulus (GPa)	Tensile Strength (MPa)	Fracture Strain (%)	Reference
Cortical bone	-	7–25	50–150	1–3	[23,58]
PEEK/ HA	Ball mill/ Injection molding	7.2 (30 wt% HA) 10.6 (40 wt% HA)	56 (30 wt% HA) 45 (40 wt% HA)	-	[116]
PEEK/ HA	In situ synthesis	-	106 (2.6 vol% HA) 99 (5.6 vol% HA) 75 (8.7 vol% HA)	-	[117]
PEEK/ nHA	Particles dispersion/cold compression/sintering	4.79 ± 0.16 (15.1 vol% nHA) 5.76 ± 0.09 (21.9 vol% nHA) 6.73 ± 0.12 (29.2 vol% nHA) 7.63 ± 0.09 (38.2 vol% nHA)	63.9 ± 1.8 (15.1 vol% nHA) 60.5 ± 2.2 (21.9 vol% nHA) 54.3 ± 2.7 (29.2 vol% nHA) 43.1 ± 1.5 (38.2 vol% nHA)	1.31 ± 0.07 (15.1 vol% nHA) 1.08 ± 0.05 (21.9 vol% nHA) 0.86 ± 0.03 (29.2 vol% nHA) 0.58 ± 0.04 (38.2 vol% nHA)	[58]
PEEK/ HA	Mixing/ Hot compression molding	~0.23 (5 wt% HA) ~0.45 (15 wt% HA) 0.58 (20 wt% HA)	71.46 (5 wt% HA) ~35 (15 wt% HA) ~11 (20 wt% HA)	-	[119]
PEEK/ mHA	Mixing/ Hot compression molding	~0.36 (5 wt% mHA) ~0.55 (15 wt% mHA) 0.72 (20 wt% mHA)	76.21 (5 wt% mHA) ~53 (15 wt% mHA) ~40 (20 wt% mHA)	-	[119]

**Table 5:** List of some reported studies of PEEK composites.

Nanoparticle	Processing Method	Key Finds	Reference
Nanorod HA	Particles dispersion/cold compression/sintering	The nanocomposites with 21.6 and 29.2 vol% had tensile strength and fracture strain close to the human cortical bones. Furthermore, the higher volume of nHA triggered better bioactivity and biocompatibility.	[58]
HA	Ball mill/ Injection molding	The tensile strength and elastic modulus of the composite with 30 wt% closely match these values for cortical bone. In vivo tests showed a higher bone contact for the composite compared to raw PEEK.	[116]
HA	In situ synthesis	The processing method promoted a better interfacial bonding between PEEK and HA, resulting in better mechanical properties. The PEEK/5.6 vol% of HA demonstrated desirable biocompatibility without apparent toxicity to the animal. In addition, the in vivo bioactivity showed that higher HA content promotes a faster new bone tissue growth around the implant made of PEEK/HA.	[117,122]
HA	Compression molding	The PEEK/40 vol% composite showed good biocompatibility and the compressive strength was in range with the cortical bone.	[118]
mHA	Mixing/ compression molding	The composite with 5 wt% of mHA showed higher tensile strength, 23% higher than pure PEEK. Higher growth of the bone tissue observed in the in vivo test was achieved for the same composite composition with 5 wt% of mHA.	[119]
25 wt% nHA/20 wt% CFR	Melt blending and injection molding	The ternary composite presented an elastic modulus higher than the values usually found for the PEEK/HA composites. Furthermore, the ternary composite improved biocompatibility in vitro and promoted osseointegration in vivo.	[125]
s-ZnO	Cryogenic ball-milling/compression molding	The PEEK/sZnO displayed superior stiffness and strength compared to the neat polymer and the composites with ZnO without modification. Moreover, the antibacterial activity was improved with increasing nanoparticle content.	[130]
hydroxylated ZnO	Extrusion	The hydroxylated PEEK was grafted in the carboxylated PEEK to prepare masterbatches. Then, the masterbatches were compounded with PEEK. A superior stiffness and strength were exhibited for the composites with polymer-grafted nanoparticles compared to the neat PEEK. Moreover, the antibacterial activity increased raising the nanoparticle content.	[131]
s-ZnO	Twin-screw extrusion/injection molding	The addition of s-ZnO increased the tensile strength and elastic modulus. However, the improvement in the mechanical properties was inferior to the [130] study. It can be associated with the processing method, along with the use difference of different silane coupling. Cell viability was enhanced for the PEEK/ZnO composites, as well as the antibacterial activity	[134]
ZnO	Co-rotating twin-screw extrusion	The incorporation of ZnO nanoparticles did not improve the mechanical properties. Nonetheless, a positive effect on biological performance was observed after incorporating ZnO.	[135]
TiO_2_	Planetary mixer/single-screw extrusion	The incorporation of TiO_2_ lightly increased the material’s stiffness and did not interfere with the tensile strength.	[136]

## Data Availability

The data presented in this study is available in the article.

## Abbreviations

**Description**	**Abbreviation**
316L SS	316L stainless steel
accelerated neutral atom beam	ANAB
additive manufacturing	AM
calcium	Ca
calcium metasilicate	CaSiO_3_
carbon fiber reinforced	CFR
computed tomography	CT
fused deposition modeling	FDM
hydroxyapatite	HA
metal-on-metal	MoM
metal-on-polymer	MoP
modified HA	mHA
nanorod HA	nHA
poly(ether-ether-ketone)	PEEK
silanized zinc oxide	s-ZnO
simulated body fluid	SBF
strain energy density	SED
strontium	Sr
tantalum	Ta
titanium	Ti
titanium dioxide	TiO_2_
ultra-high molecular weight polyethylene	UHMWPE
ultraviolet	UV
zinc oxide	ZnO
